# Personal Protective Equipment and Donning and Doffing Techniques in the Cardiac Catheterization Laboratory During the COVID-19 Pandemic: Insights From an Internet Search for Protocols

**DOI:** 10.3389/fcvm.2021.652298

**Published:** 2021-05-13

**Authors:** Justin Haloot, Omar Sheikh, Fatima Dollar, Heta Javeri, Hendre Jeannetta Fichardt, Gail Fernandes, Marlene Garcia, Anand Prasad

**Affiliations:** ^1^Division of Cardiology, Department of Medicine, The University of Texas Health Science Center at San Antonio, San Antonio, TX, United States; ^2^Division of Infectious Disease, Department of Medicine, The University of Texas Health Science Center at San Antonio, San Antonio, TX, United States

**Keywords:** COVID-19, personal protective equipment, cardiac cath lab, donning and doffing process, protocol

## Abstract

**Background:** Due to the ongoing coronavirus disease 2019 (COVID-19) pandemic, a need for precise donning and doffing protocols for personal protective equipment (PPE) among healthcare infrastructures is paramount. Procedures involving the cardiac catheterization laboratory (CCL) are routinely non-aerosolizing but have the potential for rapid patient deterioration, creating the need for aerosolizing generating procedures. Multiple societal and governmental guidelines on the use of PPE during medical procedures are available on Internet websites; however, there is limited literature available in peer-reviewed formats in this context. This study aims to provide an overview of current PPE donning and doffing protocols specific to the catheterization laboratory.

**Methods:** A series of internet searches regarding donning and doffing of PPE in the CCL including published articles and internet protocols were compiled and compared using Pubmed.gov, Google.com, www.twitter.com, and www.youtube.com.

**Results:** Most institutions used N95 masks, shoe covers, at least one head covering, face shield or goggles, two pairs of gloves, and inner and outer gowns. Doffing variation was greater than donning. Doffing has the potential to contaminate the healthcare worker (HCW), and therefore, this step of PPE management requires further study. Common steps in temporal priority included cleaning of gloved hands, removal of outer (or only) gown, removal of outer gloves, repeat gloved hand cleaning, removal of facial PPE last, and a final non-gloved hand cleaning.

**Conclusions:** This analysis provides a summary of commonly used practices that may be considered when designing CCL-specific PPE protocols. Analysis of consistent steps from the literature led the authors to formulate a suggested protocol for CCL HCWs when performing procedures on patients with confirmed or suspected/unknown COVID-19.

## Introduction

As the coronavirus disease 2019 (COVID-19) pandemic continues to evolve, there is an increased need for healthcare systems to manage personal protective equipment (PPE) resources (1). In addition, the highly contagious nature of the severe acute respiratory syndrome coronavirus 2 (SARS*-*CoV*-*2) virus requires healthcare workers (HCWs) to follow protocol driven use and removal of PPE. Although present for many years in the medical lexicon, “donning” and “doffing” have now entered more commonly into daily use with the COVID-19 pandemic. The terms “don” and “doff” are combinations of the English words “do” “on” and “do” “off” and trace their origins to the fourteenth century. Proper donning and doffing of PPE is paramount to reducing HCW exposure to the SARS-CoV-2 virus. Current data suggest that person-to-person transmission *via* respiratory droplets is the most common mode of infection. Surface contamination is also a concern with this virus. Several studies have now linked infections in HCWs to hospital-based exposure (2, 3). Data from a hospital experience in China found that inadequate use of hand washing and PPE was the most likely cause of nosocomial HCW infection (4).

The intersection of COVID-19 and cardiovascular disease is multifaceted, and these interactions have been outlined previously (5). HCWs in the cardiac catheterization laboratory (CCL) are at possible risk of viral exposure. Although routine cardiac catheterization is a non-aerosolizing procedure, the potential for clinical deterioration in critically ill patients makes this environment important to consider for level of recommended PPE use. Cardiopulmonary instability with need for non-invasive mechanical ventilation, intubation, or cardiopulmonary resuscitation (all aerosolizing treatments) can occur in patients with acute coronary syndromes—particularly with ST segment elevation myocardial infarction or cardiogenic shock. In these scenarios, time delays are suboptimal, making viral test results unavailable prior to arrival to the CCL.

Multiple societal guidelines and governmental agencies such as the Centers for Disease Control and Prevention in the United States have recommended the use of PPE when performing medical procedures. Hospital systems are commonly left to develop their own individual set of guidelines—often based on resource availability. These institutional protocols are generally not published in peer-reviewed formats but rather viewable or disseminated on Internet-based sites. The purpose of this study was to perform an Internet-based search for PPE protocols relevant to the protection of HCWs in the CCL and to provide a descriptive overview of current practices.

## Methods

**Figure 1:**
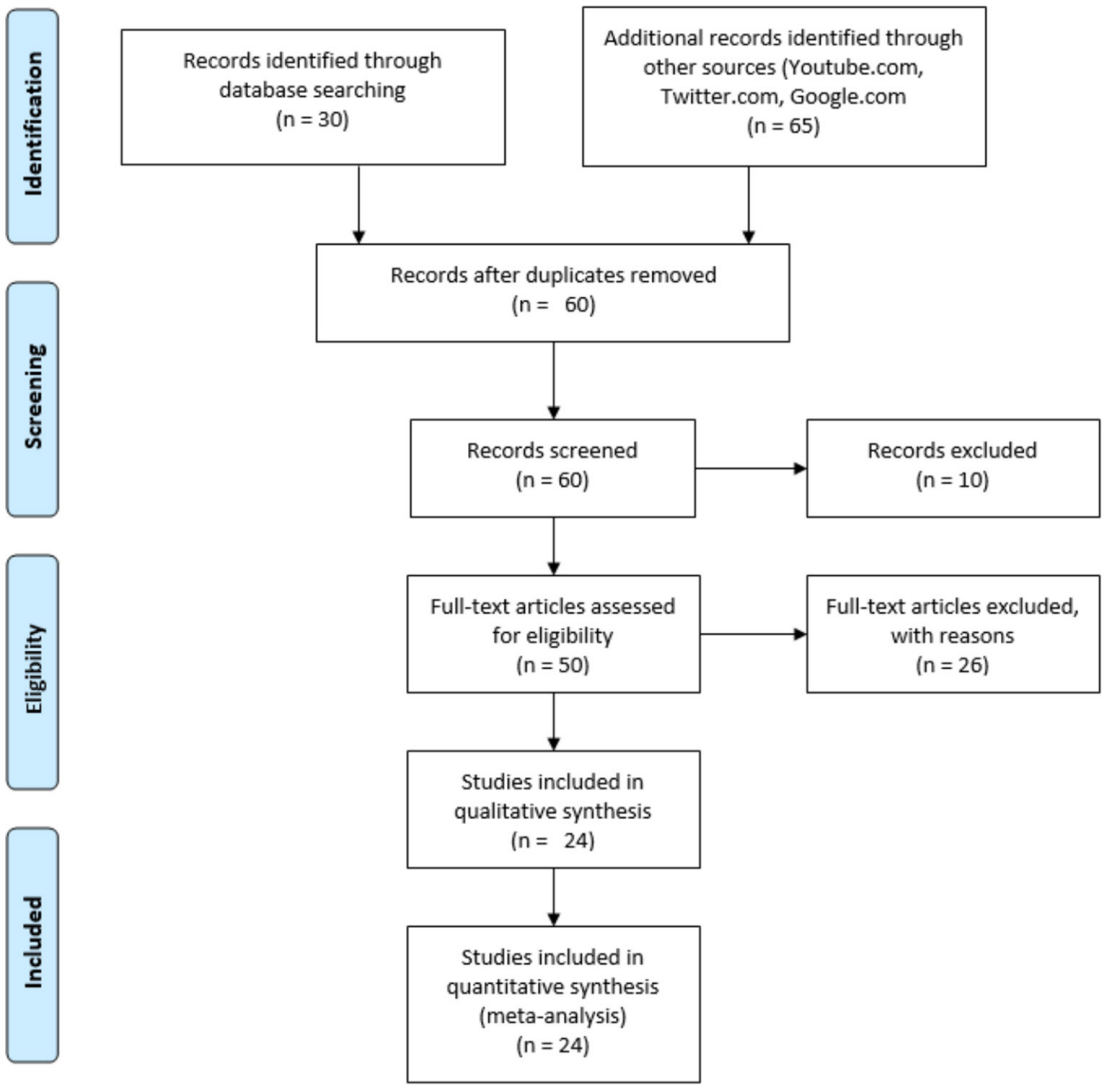
Study retrieval flow diagram.

Both published articles and protocols available on the Internet were included in the present study. For published papers, PubMed was utilized using the following terms in combinations: “cardiac catheterization,” “personal protective equipment,” and “COVID-19.” For the broader Internet search, these terms were inputted into Google.com, www.twitter.com, and www.youtube.com. The search date range for PubMed/LITCOVID was for relevant articles from January 1, 2020 through July 15, 2020, and the Internet searches were performed on October 20, 2020. Relevant articles were screened through initial PubMed review of the title, abstract, and, as needed, the full manuscript. For protocols obtained through the Internet searches, the retrieved documents were included if they contained information about both donning and doffing with associated PPE equipment delineated. The outline of the data collection searches is shown in Figure 1. The full description of each protocol from the initial web search is listed in Supplementary Table 1.

## Results

The search revealed 5 relevant articles from PubMed, 12 from Google.com searches, 1 from www.twitter.com, and 6 from www.youtube.com. Seven protocols were from individuals, and 17 were from institutions/hospitals.

### PPE Equipment

Of the protocols listed above, 24 provided granular details on equipment use, donning, and doffing. These protocols included the Society of Cardiovascular Angiography and Interventions (SCAI), Italian Society of Interventional Cardiology (GISE), European Society of Cardiology (6), Stellenbosch University and Tygerberg Academic Hospital, Indiana Chapter of ACC, Spanish Society of Cardiology, New York University (NYU), and University Health System in San Antonio, TX (UHS). We began our data analysis by looking at the equipment recommended by these protocols. The results are seen in Table 1. All protocols recommended the use of standard lead apron protection, N95 mask or equivalent, goggles and/or face shield, and two pairs of sterile gloves. One hundred percent of all protocols recommended the use of at least one head covering, with 18 (75%) recommending one head covering and 6 (20.8%) of protocols recommending two head coverings. Eight (33%) of the protocols recommended use of a surgical mask in addition to an N95.

**Table 1:** Most recommended equipment for doffing/donning PPE in cardiac cath lab.

**Equipment**	**% Recommended (%)**
Lead protection	100
N95 mask or equivalent (FFP2)	100
Goggles/face shield	100
2 pairs of sterile gloves	100
Shoe covers	83.3
1 head covering	75
2 gowns	65.2
Designated donning area	41.2
1 gown	37.5
Surgical mask (in addition to N95)	33.3
2 head coverings	20.8
1 pair of sterile gloves	0

### Donning

**Figure 2:**
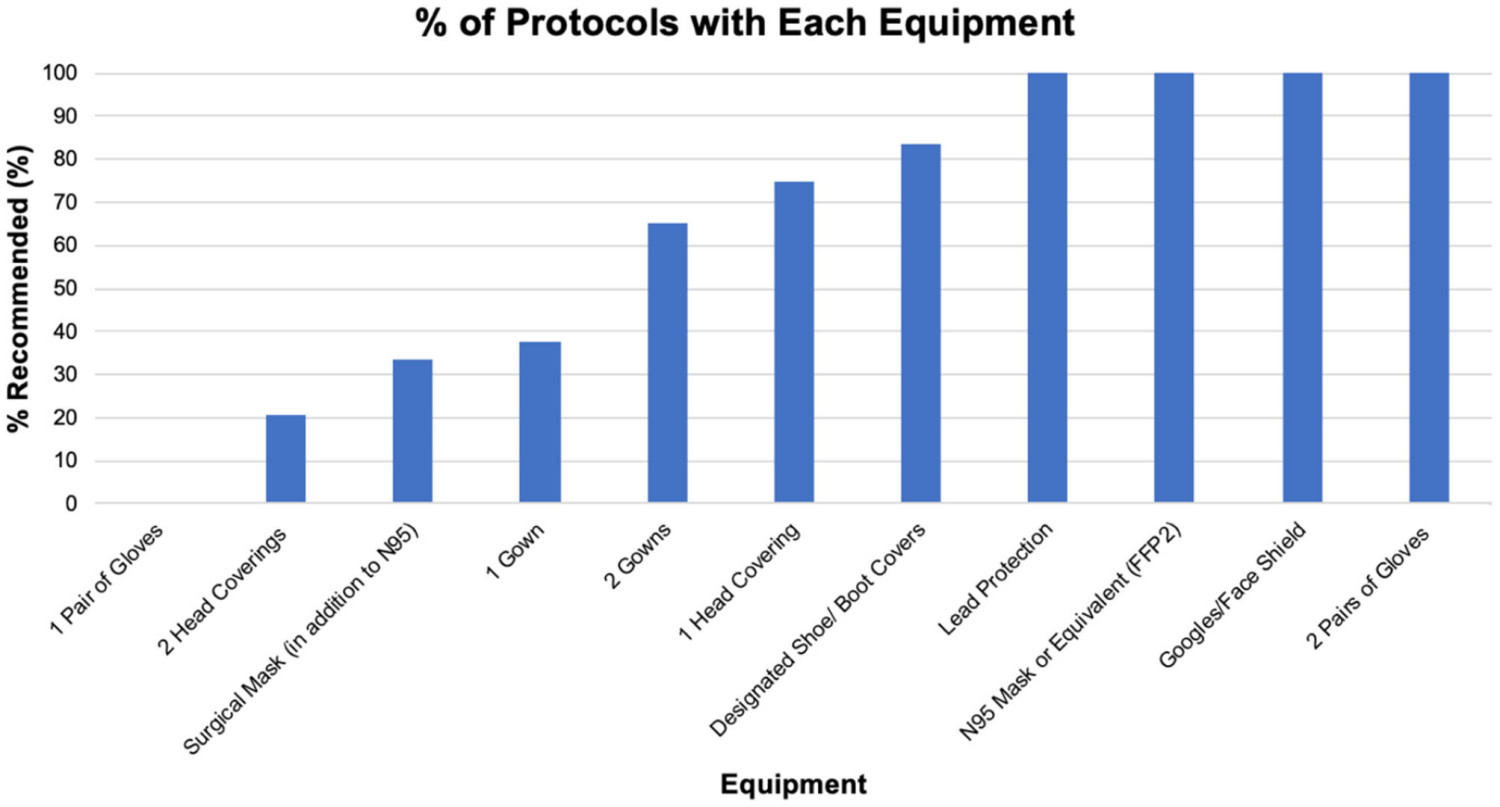
Most commonly involved steps in donning PPE.

Figure 2 demonstrates the most common steps involved in the donning of PPE before a procedure in the CCL. The particular sequence of steps in the donning process was variable among the listed protocols; therefore, instead of including a sequence, we instead constructed a figure demonstrating the most common steps involved. The x-axis shows the various steps in donning, and the y-axis demonstrates the percentage (%) of protocols that included that specific step. If a protocol required only one gown, the step was counted in the “outer gown” since there is no other gown. Similarly, if only one head covering was required in the protocol, it was counted in the “outer head cover” category. In the donning process, only 25% of protocols had a designed donning area for PPE, which is a stark difference compared to the doffing process. Seventy-four percent of protocols designated steps inside the CCL lab and outside CCL (Supplementary Table 2). In comparison, the majority of the doffing process occurs in the CCL with a few remaining steps outside the CCL.

The majority of the steps involved includes hand washing, placement of the outer gown, placement of N95 or equivalent, placement of goggles and/or face shield, placement of an outer head cover, and placement of an inner and outer pair of gloves. Forty-one percent of protocols recommended patients wear surgical masks. The remaining 59% of protocols did not specify if patients should wear masks or not; notably, no institution recommended against the patient wearing a mask. Protocols had various recommendations regarding patients getting tested, including depending on specific indications, i.e., testing in non-critical situations. The details of each protocol are specified in Supplementary Table 3.

### Doffing

**Figure 3:**
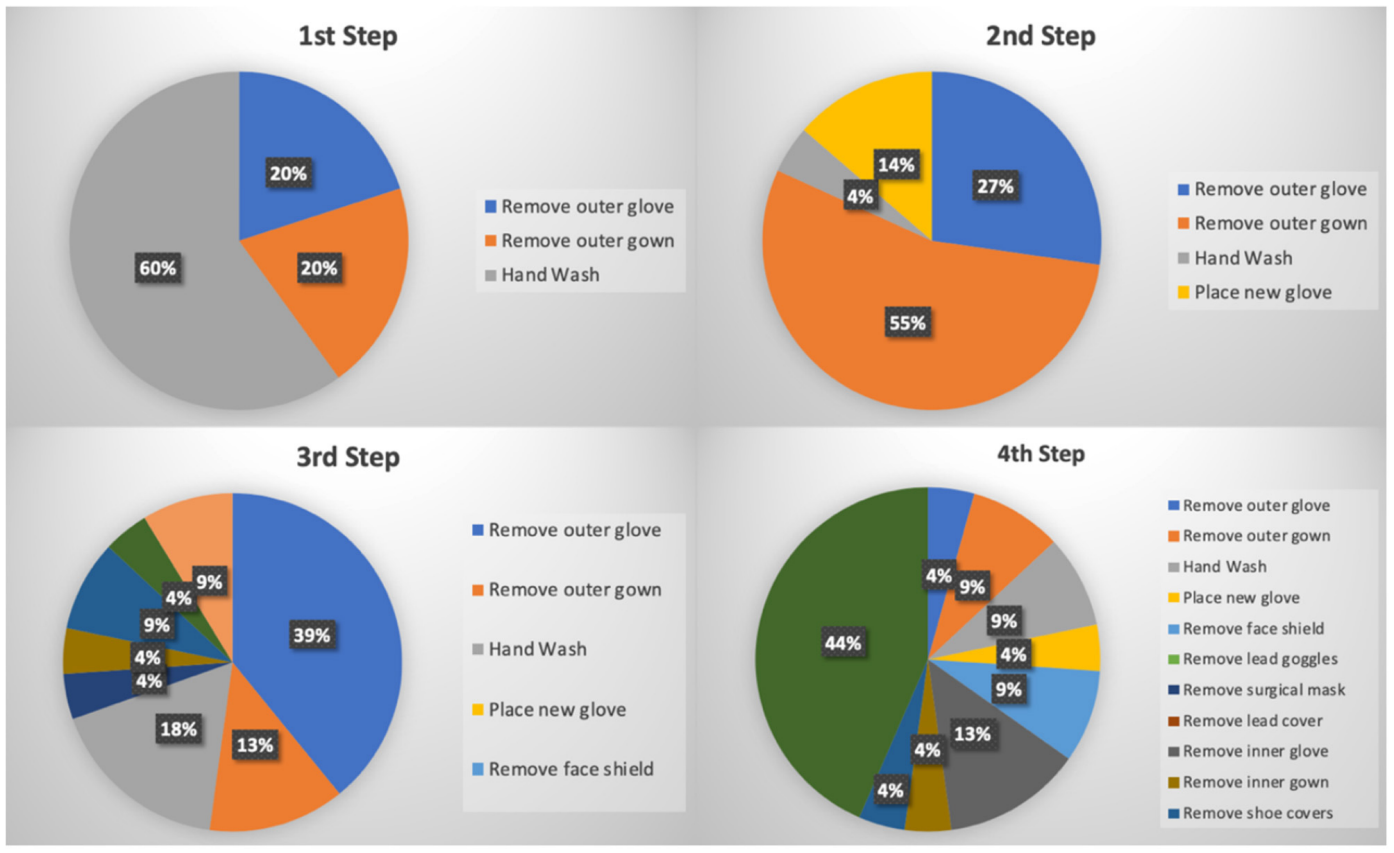
Pie chart of the first four steps recommended for doffing of PPE. Wuhan Asia Heart Hospital did not provide a doffing protocol, so the number of donning protocols = 24, and the number of doffing protocols = 23.

Doffing of PPE carries a higher risk of exposure of SARS-CoV-2 to healthcare workers after interactions with COVID-19 patients. The first four steps of the doffing process were determined to be the most crucial and noted that these steps had the most consistency (Figure 3). Across institutions, it was found that donning protocols were generally more homogeneous in regard to equipment used, whereas greater variation existed among doffing protocols. Our search indicated that hand washing was the first step in 60% of the protocols included, followed by removing outer gown (50%) as the second step. The SCAI Emerging Leader Mentorship (ELM) protocol gave a specification to use a soap and water hand wash, while the UHS protocol gave a specification of hand sanitizer (Supplementary Table 4). Other protocols did not appear to designate a specific type of hand wash. The Spanish Society of Cardiology and UHS recommended at least two hand washings—one after the removal of the outer gloves and one after the removal of the other PPE. The protocol(s) of NYU and Stellenbosch University and Tygerberg Academic Hospital had seven steps of hand washing that was done after the removal of each individual PPE (i.e., hand wash after the removal of the outer gown, hand wash after removal of the goggles/face mask, etc.). Variations began to arise starting the third step and beyond where removal of outer glove (39%), hand washing (18%), outer gown (13%), placement of a new glove (9%), and removal of the outer head cover/face shield (9%) were considered the third step. Likewise, the fourth step was also heterogeneous among institutions with removal of lead googles (44%), removal of inner glove (13%), removal of outer gown (9%), hand wash (9%), removal of face shield (9%), and placement of a new glove (4%). Variations continued to exist in the subsequent steps; however, 100% of protocols indicated removal of the lead apron to be the second to last step and additional hand washing to be the final step. Seventy-five percent of the protocols have designated steps inside the CCL and steps outside the CCL (Table 2).

**Table 2:** Suggested donning/doffing protocol.

**Donning:**	
Step 1:	Apply shoe cover
Step 2:	First hand wash
Step 3:	Wear head cover
Step 4:	Wear surgical mask
Step 5:	Wear goggles
Step 6:	Wear face shield
Step 7:	Wear lead apron
Step 8:	Sterile hand wash/solution
Step 9:	Wear sterile gown
Step 10:	Wear inner gloves
Step 11:	Wear outer gloves
**Doffing:**	
Step 1:	Hand wash with solution
Step 2:	Remove outer glove
Step 3:	Remove gown with inner gloves and then remove inner gloves
Step 4:	Wear non-sterile gloves
Step 5:	Remove lead
Step 6:	Remove goggles and face shield
Step 7:	Remove shoe covers
Step 8:	Dispose of gloves
The following occurs in COVID-19 free area	
Step 9:	Hand wash
Step 10:	Wear non-sterile gloves
Step 11:	Remove head cover
Step 12:	Remove mask and dispose or recycle
Step 13:	Hand wash

## Discussion

There has been no established, unified CCL protocol based on evidence-based medicine to protect against transmission of SARS-CoV-2. Even prior to the COVID-19 pandemic, recommendations for sterile techniques within the CCL are not supported by robust prospective clinical trials (7). Our main objective was to examine the variation in PPE used and the donning and doffing protocols available through formal and informal published sources.

General PPE use as described by the CDC first begins with proper hand hygiene before patient contact for all healthcare workers. It is recommended to use an alcohol-based hand rub with 60–95% alcohol or to wash hands with soap and water for at least 20 s, the latter being the preferred method. Following that, the isolation gown, filtering facepiece respirator or higher, face shield or goggles, and reperforming hand hygiene before putting on gloves is performed in that order. The appropriate sequence for donning and doffing PPE can be found on the CDC website: (https://www.cdc.gov/hai/pdfs/ppe/ppe-sequence.pdf).

### PPE and Infection Control Specific to the CCL

The CCL requires the use of additional radiation-specific PPE including a one- or two-piece lead/lead equivalent apron, thyroid collar, and often goggles. As noted earlier, many of the patients coming to the CCL may be unstable or may become unstable due to cardiopulmonary compromise. Conversion from contact and droplet-only PPE to aerosol protection may be impractical while managing patients in extremis. The CCL environment often includes multiple HCWs interacting with the patient including physicians, nurses, technologists, and respiratory therapists. Having optimally protective PPE for each staff member at the outset of the case is therefore helpful when dealing with suspected and confirmed COVID-19 patients.

Furthermore, although not specifically addressed in the present analysis, the risk of aerosol-mediated contamination of CCL surfaces is a concern. For this possibility, we have developed a number of interventions at our own institution that can be considered. These include using a COVID-19-specific room for procedures, minimizing extraneous equipment or supplies in the room, covering equipment with protective drapes if they cannot be relocated, decreasing unnecessary traffic into or out of the room, wiping off lead aprons with disinfectant wipes at the end of the case, use of lead PPE specific for COVID-19/potential cases, use of ultraviolet-light-based cleaning robots (Xenex, San Antonio, Texas), and a full terminal cleaning of the room. CCLs are typical divided into two primary sections: the actual procedural room and a control room. The infection control protocols described in the literature focus largely on the actual procedure room. Given that air flow is generally in continuity between the two rooms, droplet protection at a minimum with surgical masks is reasonable while in the control room. Factors such as proximity of the HCWs in the control room to the patient, airflow patterns/handling, and aerosol status of the patient may dictate the use of more protective masks (N95 respirators). The cleaning of non-disposable PPE, procedure and control rooms, and other equipment was not consistently reported in the sources from our survey. Although beyond the scope of the present analysis, HCW to HCW spread in the CCL is also a concern. Use of general hand washing, face covering, and social distancing is advised. This latter component is particularly important in break rooms where masks might be removed during mealtimes. Staggering breaks and finding additional locations for meals are often required in this context.

### Summary Recommendations

It should be emphasized that the efficacy and validity of many of the interventions described in this paper remain to be confirmed from a microbiological standpoint. The specific protocols used by individual hospitals are generally based on local infection control departments with reference to published CDC recommendations. The present analysis provides a summary of commonly used practices across multiple institutions that should be considered when designing a CCL PPE protocol. The majority of institutions used N95 masks, shoe covers, at least one head covering, face shield or goggles, two pairs of gloves, and inner and outer gowns. Doffing variation was greater than donning. Doffing has the potential to contaminate the HCW, and therefore, this step of PPE management requires further study. Common steps in temporal priority included cleaning of gloved hands, removal of outer (or only) gown, removal of outer gloves, repeat gloved hand cleaning, removal of facial PPE last, and a final non-gloved hand cleaning.

Using many consistent items and steps from the literature, a suggested protocol by the authors can be found in Table 2. Of note, after initially using two sets of gowns—outer sterile and an inner non-sterile “bunny suit”—we have adopted a single sterile outer gown strategy. This modification was done after consultation with our local infection control team due to concerns that removal of the inner full body gown would increase doffing contamination risk. In our institution, the protocol we describe is invoked for patients with COVID-19, suspected COVID-19, or those who have an unknown status of infection (suspected or not suspected). Provided that adequate PPE supplies remain, this approach affords defined protection for our CCL HCWs. We further augment our safety with universal testing of outpatients prior to catheterization procedures. Inpatients also are universally tested; however, urgent cases are done with PPE protection even if results are not available. In addition, we recommend involved HCWs to undergo simulation training per their institution's protocol to avoid or minimize exposure to infectious material.

With this study, we present a review of the current methods utilized for donning and doffing of PPE in the CCL. To date, there has been no established study formally examining the best method of donning and doffing of PPE in the CCL for the protection of HCW. This study provides an initial analysis and evidence of current practices.

## Limitations

One major limitation of this study is the utilization of atypical research methods. COVID-19 is a rapidly growing entity creating a need for rapid development of guidelines and protocols for the treatment of these patients. Our research depended on protocols published by the various institutions in multiple platforms on the Internet including YouTube and Twitter. The amount of information is dependent on the amount shared by each institution, which makes it difficult to compare. It was noted that some institutions also promoted similarly used content. For example, cardiovascular innovations utilized similar material from GISE. We counted the protocol as a protocol for cardiovascular innovations, as this was what they recommended publicly. Therefore, there may be some duplicate protocols due to being similar to other published information.

Currently, there is a lack of randomized studies to determine which specific protocol would provide the best protection for healthcare workers. Our study is mainly descriptive in nature. This is largely in part due to the diverse nature of the protocols utilized at the different institutions. This would make it difficult to allow for comparison. We recommend that institutions develop a trial to study their specific protocol and examine the rate of COVID-19 transmission to HCWs in the CCL and compare their results with other institutions with the ultimate goal of identifying practices that decrease COVID-19 transmission.

Despite these limitations, media outlets have allowed for greater communication of protocols between institutions. Rather than waiting for a unified guideline, institutions can share their experiences in real time. Shared information has allowed for the formation of more formal statements/guidelines as seen by SCAI, GISE, European Society of Cardiology (ESC), and the Spanish Society of Cardiology.

The fight against COVID-19 and PPE continues to evolve, which provides a temporal limitation to our study. As the pandemic continues, the medical community innovates in PPE utilization. One current new method is the use of antibacterial and antiviral agents for the reuse of PPE. The most promising methods include ultraviolet germicidal irradiation, vaporous hydrogen peroxide, and moist heat. The CDC recommends utilization of these methods mainly when there is a shortage of PPE or filtering facepiece respiratory (FFR) (8). More studies will be needed to determine the efficacy of these methods.

## Conclusions

As the COVID-19 pandemic evolves, the protection of healthcare workers has become crucial. HCWs can significantly reduce their risk of acquiring the virus by adhering to society/guideline recommendations, specifically during the donning and doffing of PPE. The steps involved in the donning and doffing vary across institutions. From our analysis, we found 24 protocols from multiple databases including PubMed, Google.com, www.twitter.com, and www.youtube.com, listing the number of steps involved and the amount of PPE used per protocol. We have a suggested protocol that we developed at our home institution, University Health System, San Antonio. With time and more analysis, our hope is that a unified protocol that carries the lowest risk of contamination for HCWs can be adopted among many institutions.

## Data Availability Statement

The original contributions presented in the study are included in the article/Supplementary Material, further inquiries can be directed to the corresponding author.

## Author Contributions

All authors listed have made a substantial, direct and intellectual contribution to the work, and approved it for publication.

## Conflict of Interest

The authors declare that the research was conducted in the absence of any commercial or financial relationships that could be construed as a potential conflict of interest.
