# Supplementary material for: Personal Protective Equipment and Donning and Doffing Techniques in the Cardiac Catheterization Laboratory During the COVID-19 Pandemic: Insights From an Internet Search for Protocols
Source: Front Cardiovasc Med. 2021 May 13;8:652298. doi: 10.3389/fcvm.2021.652298 (PMC8155273; doi:10.3389/fcvm.2021.652298)
Supplement: Supplementary file 1 [file Data_Sheet_1.docx]

**Supplemental Table 1. Detailed description of each individual protocol from the initial web search**

| **Article/ Institution** | **Author** | **Source (Twitter, journal, website, etc.)** | **Recommendations/ Protocol** |
| --- | --- | --- | --- |
| Cardiovascular Innovations Foundations | Google  Giuseppe Tarrantini | COVID19 Cath Lab Implications | Donning  1. Remove any personal items  2. Put on the lead apron  3. Put on a first disposable gown  4. Gather the necessary PPE and check for their integrity  5. Perform hand hygiene with alcohol hand gel/rub  6. Put on the proper disposable respirator N95 or FFP2 standard  (FFP3 available for anesthesiologist and nurse helping on airways  maneuvers)  7. Put on hair cover  8. Put on shoe covers  9. Put on googles and/or face shield avoiding any interference with  the respirator  10. Perform hand hygiene  11. Put on the first pair of gloves  12. Put on a gown (sterile or not according to your role in cath lab)  not using the inside tie  13. Put on a second pair of gloves (over cuff), sterile if needed.  Doffing  Inside the operating room:  a. Wait until patient is out of the room; close the door.  b. Perform hand hygiene over the gloves.  c. Peel off gown and gloves together and roll inside, slowly and carefully,  avoiding aerosolization.  d. If gloves are removed separately, touch only the external part (use  glove-in-glove or beak technique).  e. Perform hand hygiene (over the internal gloves).  f. Remove face shield and/or googles avoiding contact with face and  eyes and dispose them safely or put in a separate container for  reprocessing.  g. Perform hand hygiene (over the internal gloves).  h. Remove hair cover and dispone it safely.  i. Remove shoe covers and dispone them safely.  j. Perform hand hygiene (over the internal gloves).  k. Remove internal gloves and dispose them safely.  l. Perform hand hygiene.  m. Step out of the operating room and immediately close the door.  Outside the operating room:  n. Put on another pair of gloves.  o. Remove facial respirator without touching the front side of the  respirator.  p. Remove the disposable gown and the gloves.  q. Remove lead apron.  r. Perform hand hygiene with soap and water and alcohol gel/rub. |
| Indiana-ACC Virtual Forum Cath Lab Protocols for COVID19 | Google | Google | Donning:  1. Tall disposable shoe covers  2. Covid-19 designated lead apron.  3. Leaded glasses or prescription glasses  4. First head cover (cover ears)  5. N95 Mask  6. Second head cover (cover ears)  7. PPE Mask – N95, etc  8. Eye protection: Goggles or face shield  9. Hand Hygiene: Surgical scrub  10. Non-sterile gown (Don’t tie back?)  11. Sterile gloves 1  12. Sterile gown  13. Sterile gloves 2  Doffing:  1. Hand Hygiene (HH1)  2. Remove surgical gown by breaking neck/back straps and dispose sterile gloves. (Don’t tie to begin with?)  3. HH2 with alcohol foam in room  4. Remove eye protection  5. Remove surgical mask  6. Remove second head cover in room  7. Remove PPE gown and gloves  8. HH3 with alcohol foam in room  9. Remove shoe covers at doorway in room and step out of room  10. HH4 with alcohol-based disinfectant (i.e.: Sterillium)  11. Remove N95 mask  12. Remove first head cover  13. HH5 with surgical scrub 1  14. Remove Covid-19 lead  15. Change to clean scrubs |
| CSANZ Consensus Guideline for Intervetional Cardiology Services Delivery in Australia and New Zealand | Lo Sith, Young | Google | (order as in the Spanish Society of Cardiology guidelines)  Dressing  Outside the lab  No jewelry  Tie hair back  Lead apron  Correct Hand Hygiene  FFP2 mask  Splash goggles  Cap  Hand washing  First pair of gloves  Put on the gown  Inside the Lab  Roll second pair of gloves over the first  Undressing  Inside the lab  Remove the gown and outer pair of gloves  Outside the lab  Remove cap  Peel off second pair of gloves  Wash hands  Remove goggles with eyes closed  Remove mask by lifting elastic bands  Wash hands |
| NYU | Google  Sripal Bangalore | <https://drive.google.com/file/d/1wPb4eXx1tW5MeuczlZSaxVgWsMBgsqKc/view> | Donning  Hand Wash  Boot covers and Lead Apron  Disinfect hands  N95 mask, cap, surgical mask  Goggles/ Face Shield  Disinfect hands  1st pair of gloves  Enter cath lab  Gown  2nd pair of gloves  Doffing  Disinfect  Remove outer gown and outer gloves  Disinfect gloves  Remove boot covers  Disinfect gloves  Remove gloves  Disinfect hands  Exit lab  Remove face shield, cap, N95 mask  Final hand washing |
| Donning/Doffing PPE for angiography during COVID19 Crisis | Google  John TJ | European Heart Journal (2020) 0, 1-2 | **Donning of PPE**   1. Surgical scrubs to be worn. These will need to be changed with each case if a COVID-positive case is treated or there is any concern about soiling. No jewelry to be worn. Scrub top to be tucked into scrub pants. 2. Thorough washing of hands for at least 20 seconds prior to initiating the donning process. 3. Shoe covers to be initially donned. 4. Thereafter, prior to scrubbing, don the N95 respirator mask with the surgical scrub cap over the N95 mask. A second method for eye protection should be used and worn over the respirator and scrub cap. This protocol recommends a surgical mask with a visor be used for eye protection. Disposable face shields may also be used. Surgical safety goggles can be used as an alternative but will need to be sterilized after each case. Healthcare workers should ideally have N95 respirator masks fit-tested and the correct size respirator mask should be used. 5. The scrub cap should cover both ears. 6. Prescription glasses/lead glasses on their own are not enough for protection. The visor should adequately cover both eyes and provide some protection to the side of the face. Put on usual radiation protection equipment as used. 7. Thoroughly scrub hands to the level of the elbows using an antibacterial surgical scrub and warm water and prepare to gown. Disposable surgical gown to be worn. Take care to ensure that gown sleeves do not go beyond wrists as it is put on. Ensure that the top of the gown covers as much of the exposed neck area as possible 8. This hospital protocol recommends a two-glove technique. Put on two pairs of gloves ensuring that both gloves fit snugly and cover the sleeves of the gown.   **Doffing of PPE**   1. There is a very high risk of contamination and therefore another individual should watch you doffing the PPE and alert you to any possibility of contamination. 2. Wash/clean the outer pair of gloves in water and thereafter remove the outer pair without touching the outside surface of the inner pair of gloves, and discard into a medical waste bin. 3. Remove shoe-covers and discard (performed more easily if sitting on a chair). 4. There is contention as to whether one should wash hands/gloves with alcohol during each step of doffing. This institution recommends cleaning hands between every step of the procedure. 5. Clean internal pair of gloves with alcohol. 6. Remove the visor without touching the front/exposed surface of the visor and dispose directly into medical waste bin. 7. Clean internal pair of gloves with alcohol (again – second time). 8. Remove the disposable gown by grasping the inside surface of the gown at the collar and rolling the gown away from you without touching the exposed surface of the gown. 9. Clean internal pair of gloves with alcohol (again – third time). 10. Remove scrub cap. 11. Clean internal pair of gloves with alcohol and then remove (fourth and final alcohol clean of internal gloves). 12. Remove radiation protection. 13. Clean hands with alcohol. 14. Remove N95 respirator mask by pulling the elastics over one’s head and discard the mask without touching the exposed surface of the mask. The respirator is removed last to reduce the likelihood of exposure to aerosolized droplets during doffing. 15. Wash hands with soap and warm water. 16. Radiation protection should be cleaned with an alcohol scrub, surgical scrubs should be washed, and the chair used during doffing of overshoes should be cleaned with an alcohol scrub. 17. There is a recommendation that you then take a shower, but the operators at this hospital aim to shower only before leaving the hospital. |
| Consensus Document of Interventional Cardiology and Heart Rhythm Association of the Spanish Society of Cardiology | Google  Rafael-Romaguera et. al. | REC Interv Cardiol 2020 | Dressing  Outside the lab  No jewelry  Tie hair back  Lead apron  Correct Hand Hygiene  FFP2 mask  Splash goggles  Cap  Hand washing  First pair of gloves  Put on the gown  Inside the Lab  Roll second pair of gloves over the first  Undressing  Inside the lab  Remove the gown and outer pair of gloves  Outside the lab  Remove cap  Peel off second pair of gloves  Wash hands  Remove goggles with eyes closed  Remove mask by lifting elastic bands  Wash hands   - Patients: surgical mask. It is important to stress that FFP2 masks are personal protection masks and not barrier masks. The air expelled by these masks is contaminated and so they should not be worn by infected patients. Patients should wear a face mask that acts as a barrier to secretions. - Physicians and nurses: hand-washing, coated fluid-impermeable gown with cuff (if the gown is not fluid-impermeable, a plastic apron should be added), 2 pairs of gloves (whose use is recommended by some local authorities), splash goggles or conventional goggles and face shield, cap, and high filtration efficiency FFP2 mask if available[^4^](https://www.recintervcardiol.org/en/?option=com_content&view=article&id=328&catid=40#ref4) (for procedures such as placement of implantable cardioverter-defibrillators, pacemakers and transcatheter prostheses, a surgical mask should be placed over the FFP2 mask). Closed work shoes are recommended or, if unavailable, boots. - Cardiologists or circulating nurses: gloves, cap, fluid-impermeable gown and FFP2 face mask (if available). |
| Considerations for cardiac catheterization laboratory procedures during the COVID‐19 pandemic perspectives from the Society for Cardiovascular Angiography and Interventions Emerging Leader Mentorship (*SCAI ELM*) Members and Graduates | Google  Molly Szerlip et al | Catheter and Caridovascular Interventions | Donning  1. Tall disposable shoe covers  2. Covid‐19 designated lead apron  3. Leaded glasses or prescription glasses  4. First head cover (cover ears)  5. N95 mask  6. Second head cover (cover ears)  7. Surgical mask  8. Eye protection: Goggles or face shield  9. Hand hygiene: Surgical scrub  10. Nonsterile gown  11. Sterile gloves 1  12. Sterile gown  13. Sterile gloves 2  Doffing:  1. Hand hygiene (HH1)  2. Remove surgical gown by breaking neck/back straps and dispose sterile gloves  3. HH2 with alcohol foam in room  4. Remove eye protection  5. Remove surgical mask  6. Remove second head cover in room  7. Remove personal protective equipment gown and gloves  8. HH3 with alcohol foam in room  9. Remove shoe covers at doorway in room and step out of room  10. HH4 with alcohol‐based disinfectant (i.e., Sterillium)  11. Remove N95 mask  12. Remove first head cover  13. HH5 with surgical scrub  14. Remove Covid‐19 lead  15. Change to clean scrubs |
| Wuhan Asia Heart Hospital | Google  Chengyi Xu | <https://rutherfordmedicine.com/videos/Ppe-Requirements-For-Covid-19-From-Wuhan-A752BEB9A?tracking_id=4B2D3F47-7439-4117-B8F1-CDF43EB89790> | **COVID19 with ACS for Emergency PCI**  Designated Cath Room  1. Designated cath lab for COVID19 pts  2. Close air conditioning, maintain fresh air system and air purifier in a continuous open state  3. Protection for DSA arm and detector  4. Yellow double-layer garbage bag set for medical garbage  5. Prepare all kinds of consumables that may be used in the operation  1 operator, 1 assistant, 1 nurse, 1 technician  Grade II or above PPE during pt transfer  Wearing Order  1. Hand Hygiene  2. Wash clothes  3. Mask, surgical cap  4. Lead Coat  5. Hand hygiene  6. Protective clothing  7. Shoe covers  8. Goggles/ Face Shield  9. Hand Hygiene (quick disinfectant)  10. Surgical Gown  11. Double Gloves  Pt transfer into Cath Lab  Pt wears a mask (preferably N95) and a hat during transfer  Under protection of Grade II or above  Grade I Protection  Isolation Gown  Hand Hygiene  Surgical Cap  Wear Mask  Grade II Protection  Grade I protection and then the following:  Put on protective clothing  Wear Gloves  Wear Goggles  Shoe Covers  Grade III Protection  All of the above + electric air supply filter respirator (positive pressure head cover) |
| Italian Society of Interventional Cardiology (GISE) position paper for Cath lab‐specific preparedness recommendations for healthcare providers in case of suspected, probable or confirmed cases of COVID‐19 | Google |  | DONNING PPE SEQUENCE     1. Remove any personal item 2. Put on the lead apron 3. Put on a first disposable gown 4. Gather the necessary PPE and check for their integrity 5. Perform hand hygiene with alcohol hand gel/rub 6. Put on the proper disposable respirator N95 or FFP2 standard (FFP3 available for anaesthesiologist and nurse helping on airways manoeuvres) 7. Put on hair cover 8. Put on shoe covers 9. Put on googles and/or face shield avoiding any interference with the respirator 10. Perform hand hygiene 11. Put on the first pair of gloves 12. Put on a gown (sterile or not according to your role in cath lab) not using the inside tie 13. Put on a second pair of gloves (over cuff), sterile if needed.         DOFFING PPE SEQUENCE    Inside the operating room (or in the filter zone if available):   1. Wait until patient is out of the room; close the door. 2. Perform hand hygiene over the gloves 3. Peel off gown and gloves together and roll inside, slowly and carefully, avoiding aerosolization 4. If gloves are removed separately, touch only the external part (use glove-in-glove or beak technique) 5. Perform hand hygiene (over the internal gloves) 6. Remove face shield and/or googles avoiding contact with face and eyes and dispose them safely or put in a separate container for reprocessing 7. Perform hand hygiene (over the internal gloves) 8. Remove hair cover and dispone it safely 9. Remove shoe covers and dispone them safely 10. Perform hand hygiene (over the internal gloves) 11. Remove internal gloves and dispose them safely 12. Perform hand hygiene 13. Step out of the operating room and immediately close the door.     Outside the operating room: 14. Put on another pair of gloves 15. Remove facial respirator without touching the front side of the respirator 16. Remove the disposable gown and the gloves 17. Remove lead apron 18. Perform hand hygiene with soap and water and alcohol gel/rub. |
| ACC: donning and doffing of PPE for angiography: -Protocol used during covid crisis from Tygerberg Hospital and Stellenbosch University Cape Town, South Africa | **Google**  John TJ, Hassan K, Weich H. | ACC: Tygerberg Hospital and Stellenbosch University Cape Town, South Africa | **Donning of PPE**   1. Wear surgical scrubs, change with each case. Tuck scrub top into pants. Remove all jewelery prior to donning PPE 2. Wash hands for 20 seconds, wear shoe covers 3. Wear n95 respirator with surgical scrub cap over N95 mask. Secondary eye protection should be used over the respirator and scrub cap-they specifically recommend surgical mask with visor or disposable face shields. If using surgical goggles, will need to be sterilized after each case. 4. Scrub cap should cover B/L ears 5. Prescription/Lead glasses should be covered with visor. When wearing disposable gown, gown sleeves should not go beyond the wrists. Wear two gloves   **Doffing of PPE**   1. There is a very high risk of contamination and therefore another individual should watch you doffing the PPE and alert you to any possibility of contamination. 2. Wash/clean the outer pair of gloves in water and thereafter remove the outer pair without touching the outside surface of the inner pair of gloves, and discard into a medical waste bin. 3. Remove shoe-covers and discard (performed more easily if sitting on a chair). 4. There is contention as to whether one should wash hands/gloves with alcohol during each step of doffing. This institution recommends cleaning hands between every step of the procedure. 5. Clean internal pair of gloves with alcohol. 6. Remove the visor without touching the front/exposed surface of the visor and dispose directly into medical waste bin. 7. Clean internal pair of gloves with alcohol (again – second time). 8. Remove the disposable gown by grasping the inside surface of the gown at the collar and rolling the gown away from you without touching the exposed surface of the gown. 9. Clean internal pair of gloves with alcohol (again – third time). 10. Remove scrub cap. 11. Clean internal pair of gloves with alcohol and then remove (fourth and final alcohol clean of internal gloves). 12. Remove radiation protection. 13. Clean hands with alcohol. 14. Remove N95 respirator mask by pulling the elastics over one’s head and discard the mask without touching the exposed surface of the mask. The respirator is removed last to reduce the likelihood of exposure to aerosolized droplets during doffing. 15. Wash hands with soap and warm water. 16. Radiation protection should be cleaned with an alcohol scrub, surgical scrubs should be washed, and the chair used during doffing of overshoes should be cleaned with an alcohol scrub. 17. There is a recommendation that you then take a shower, but the operators at this hospital aim to shower only before leaving the hospital. |
| University Hospital San Antonio |  |  | **Donning PPE for a Heart Alert Patient with Airborne and Droplet Precautions**   1. Wear UHS laundered scrubs 2. Perform hand hygiene 3. Put on shoe covers (these are not considered PPE)    1. Consider using a pair of shoes that you only use at the hospital and leave them here 24/7 4. Put on N95 mask - a second mask is not required 5. Put on bonnet (this is not considered PPE) - no knots in the ties 6. Put on any lead equipment you will be using 7. Put on lead googles or disposable goggles 8. Put on a disposable face shield 9. Put on a gown over your lead -- this is your PPE 10. Put on gloves (2 pair as needed) 11. Gloves should cover wrist of gown   **Doffing of PPE in Preparation to Transport Patient from the Cath Lab**   1. Move to the doffing area inside the Lab 2. Remove and discard contaminated gloves and gown 3. Use hand sanitizer 4. Put on non-sterile gloves if you have bare hands 5. Remove and discard face shield- do not touch the front    1. Do not remove N95 mask yet 6. Remove lead goggles place on lead rack - do not touch the front of the items 7. Take off lead from chin down and hang on lead rack - do not touch the front of the lead 8. Remove and discard gloves 9. Apply clean gloves 10. Wipe off your lead items with Sani-Wipes and leave lead on lead rack to be   Xenexed   - 1. Lead should be wet for 2 minutes or per manufacturer’s instructions  1. Move to doffing area in the Lab 2. Remove booties, then gloves and discard in the Lab 3. Leave on goggles and N95 mask for transporting the patient 4. Apply hand sanitizer or wash your hands 5. For transporting the intubated patient put on a yellow gown, a clean face shield   and clean gloves and prepare to transport the patient   1. If you are not transporting the patient wash your hands 2. Proceed with N95 mask procedure for recycling |
| Hammersmith Hospital | Youtube | <https://www.youtube.com/watch?v=aP_7NBaPq5E> | Donning PPE @ designated donning area   1. Placing shoes/boots 2. Place lead apron 3. Wash hands 4. Surgical cap/ hood 5. N95/ FP3 mask 6. Visor/ Face Shield 7. Wash Hands 8. Inner gown 9. Inner glove 10. Wash Hands (with gloves on) 11. Sterile outer gown 12. Sterile outer gloves   Doffing in the lab   1. Remove face shield/ visor 2. Remove outer gloves 3. Remove outer gown 4. Remove inner gown 5. Remove inner gloves 6. Remove N95/FP3 mask 7. Remove surgical cap/ hood   Doffing in designated area out of lab   1. Clean shoes/boots 2. Remove shoes/ boots 3. Remove lead 4. Wash hands 5. Change clothes 6. Wash hands |
| London Cardio Clinic | Youtube | <https://www.youtube.com/watch?v=AXTDWbnHXIk> | Donning@ designated area   1. Inner head cover 2. Remove badges/ pens 3. N95 mask 4. Goggles 5. Outer head cover 6. Visor/ Face shield 7. Boots/ shoes 8. Lead coverings 9. Wash hands 10. Inner gown 11. Wash hands (sterile) 12. Inner gloves 13. Outer gown 14. Outer gloves |
| Immad Sadiq, D (Hartford, CT) | Youtube | <https://www.youtube.com/watch?v=g0_iZnPrM1k> | Donning @ Clean Zone   1. Remove badges 2. N95 mask 3. Bouffant (head covering) 4. Shoe coverings 5. Long pair shoe covering 6. Outer head covering (hood) 7. Surgical Mask 8. Face shield   Donning @ Cold Zone   1. Hand Wash (with sanitizer) 2. Place lead 3. Inner gown 4. Inner sterile gloves   Donning @ warm zone   1. Wash hands (with sanitizer) 2. Outer sterile gown 3. Outer sterile gloves   Doffing @ hot zone (in the cath lab)   1. Place a third pair of sterile gloves 2. Remove face shield 3. Remove outer mask 4. Wash hands 5. Remove outer head covering 6. Wash hands 7. Remove outer boot/ shoe covering 8. Wash hands 9. Remove outer most layer of gloves 10. Wash hands 11. Remove outer gown 12. Remove outer gloves   Doffing @ warm zone   1. Wash hands 2. Remove inner gown 3. Remove inner gloves   Doffing @ cold zone   1. Wash hands 2. Place new gloves 3. Remove lead 4. Remove gloves 5. Wash hands   Doffing @ clean zone   1. Place new gloves 2. Remove inner head covering (bouffant) 3. Remove inner shoe covers 4. Remove gloves 5. Wash hands 6. Place new gloves 7. Remove N95 |
| St. George’s Hospital, London, UK | Youtube | <https://www.youtube.com/watch?v=QrVHDCJpPA4>  <https://www.youtube.com/watch?v=oy4xTRkm_Zg> | Donning outside cath lab   1. Place lead 2. Apron over lead 3. Wash hands 4. Head covering (bouffant) 5. Inner gown 6. N95 mask 7. Face Shield 8. Inner Gloves 9. Remove shoes 10. Place boot   Donning in the cath lab   1. Wash hands 2. Outer gown 3. Outer gloves   Doffing in the cath lab   1. Remove outer gown 2. Remove outer gloves 3. Remove inner gown 4. Remove inner gloves 5. Remove apron over lead 6. Wash hands 7. Place new pair of gloves 8. Clean visor 9. Remove gloves   Doffing outside cath lab   1. Remove visor 2. Remove N95 3. Remove shoes 4. Place gloves 5. Clean and remove lead 6. Remove gloves 7. Change scrubs 8. Hand wash |
| Princess Fatima Academy | Youtube | <https://www.youtube.com/watch?v=XQGmNfqJ0rc> | Donning of PPE   1. Lead apron 2. Hand wash 3. Shoe covers 4. Inner gown 5. N95/FFP2 or FFP3 mask 6. Hair cover 7. Goggles or Face Shield 8. Hand wash 9. Inner gloves 10. Outer gown 11. Outer gloves   Doffing of PPE   1. Hand Wash 2. Remove outer gown 3. Remove outer gloves 4. Hand wash 5. Remove shield/ goggles 6. Hand wash 7. Remove head cover 8. Hand wash 9. Remove mask 10. Hand wash 11. Remove inner gown 12. Remove inner gloves |
| Giuseppe Tarantini (GISE) | Youtube | <https://www.youtube.com/watch?v=C83AHSjcrvw&feature=youtu.be> | Donning of PPE   1. Place lead 2. Hand Wash 3. Shoe Covers 4. Inner gown 5. N95/FFP2 or FFP3 6. Head Covering 7. Goggles/ Face Shield 8. Hand Wash 9. Inner gloves 10. Outer gown 11. Outer gloves   Doffing of PPE  *In cath lab*   1. Hand wash 2. Remove outer gown 3. Remove outer glove 4. Hand wash 5. Remove face shield/ goggles 6. Hand wash 7. Remove head cover 8. Remove shoe cover 9. Hand wash 10. Remove inner glove 11. Hand wash   *Outside Cath Lab*   1. Place new gloves 2. Remove mask 3. Remove inner gown 4. Remove additional glove 5. Hand wash |
| CathLab Digest | Google | <https://www.cathlabdigest.com/content/dofficer-tips-donning-and-doffing-ppe> | Donning of PPE  1. Surgical Scrubs and Scrub cap  2. Wash hands  3. Lead protection  4. Shoe covers  5. N95  6. Inner gown  7. Inner gloves  8. Wash gloves  9. Face-shield  *In the Cath lab*  10. Outer gown  11. Outer gloves  Doffing of PPE  *Exit cath lab*  1. Remove shoe covers  2. Wash hand  3. Remove outer gown  4. Remove outer gloves  5. Wash hands  6. Remove face shield  7. Wash hands  8. Wash face shield  9. Remove N95  10. Wash hands  11. Remove scrub cap  12. Wash hand  13. Remove inner glove  14. Wash hands  15. Remove lead |
| International Journal of Cardiovascular Sciences | Google  Zeferino Mariano, G  Int. J. Cardiovasc. Sci. vol.33 no.3 Rio de Janeiro May/June 2020 Epub May 29, 2020 | <https://www.scielo.br/scielo.php?script=sci_arttext&pid=S2359-56472020000300288&lng=en&nrm=iso> | Donning PPE  *Anteroom of Cath Lab*  1. Hand wash  2. Scrub cap  3. N95/ FFP2  4. Goggles/ Face Shield  5. Shoe Covers  6. Lead Apron  7. Inner gown  8. Hand wash  9. Inner gloves  *In Cath Lab*  10. Outer gown  11. Outer gloves  Doffing PPE  *In Cath Lab*  1. Hand Hygiene  2. Remove outer gown  3. Remove outer gloves  4. Hand wash  5. Remove hair cap  6. Remove shoe cover  7. Remove goggles  8. Hand hygiene  *Outside Cath Lab (CCL anteroom*)  9. Remove inner gloves  10. Remove face shield  11. Remove N95/ FFP2  12. Hand Hygiene  13. Remove lead  14. Hand hygiene |
|  | Google | <https://www.escardio.org/Education/COVID-19-and-Cardiology/ESC-COVID-19-Guidance> | Donning  1. Place work clothes and shoes  2. Wash hands  3. Surgical Cap  4. N95 mask  5. Inner gloves  6. Goggles  7. Inner gown  8. Outer gloves  Doffing  1. Remove outer gloves  2. Place new gloves  3. Hand wash  4. Remove gown  5. Remove outer gloves  6. Hand wash  7. Remove goggles  8. Hand wash  9. Remove N95 mask  10. Hand wash  11. Remove cap  12. Hand wash  13. Remove inner gloves |
|  | Twitter  Jara et. la. | <http://www.scai.org/Press/detail/ppe-donning-doffing-in-cardiac-catheterization-lab#.Xw-sZ5NKgWp> | Donning  1. Hand hygiene  2. Shoe covers  3. Lead apron  4. Lead or prescription glasses  5. Inner head cover  6. N95 mask  7. outer head cover  8. Surgical mask  9. Goggles/ Face shield  10. Hand wash  11. Inner gown  12. Inner gloves  13. Outer gown  14. Outer glove  Doffing  1. Hand hygiene  2. Remove outer gown  3. Remove outer gloves  4. Hand Hygiene  5. Remove goggles/ face shield  6. Remove surgical mask  7. Remove outer head cover  8. Remove inner gown  9. Remove inner gloves  10. Hand Hygiene  11. Remove shoe covers  *Outside Cath Lab*  12. Hand Hygiene  13. Remove N95  14. Remove inner head cover  15. Hand Hygiene  16. Remove lead apron  17. Change scrubs |
| Bettari, et. al. | Pubmed accessed on July 15, 2020  Key terms “Cardiac Catheterization PPE” and “COVID and heart catheterization” and “COVID and cardiac catheterization”  37 results  3 with protocols after duplicates were removed | Bettari L, Pero G, Maiandi C, et al. Exploring Personal Protection During High-Risk PCI in a COVID-19 Patient: Impella CP Mechanical Support During ULMCA Bifurcation Stenting [published online ahead of print, 2020 Apr 10]. JACC Case Rep. 2020;10.1016/j.jaccas.2020.03.006. doi:10.1016/j.jaccas.2020.03.006 | Per the European CDC PPE Protocol that was followed in the paper  Donning  1. Hand Hygiene  2. Gown  3. FFP Class 2 or 3  4. Surgical Mask over FFP  5. Goggles  6. Gloves  Doffing  1. Remove gloves  2. Place on new gloves  3. Remove gown  4. Remove goggles  5. Remove FFP |
| Chieffo et. al. | Pubmed accessed on July 15, 2020  Key terms “Cardiac Catheterization PPE” and “COVID and heart catheterization” and “COVID and cardiac catheterization”  37 results  3 with protocols after duplicates were removed | Chieffo A, Stefanini GG, Price S, et al. EAPCI Position Statement on Invasive Management of Acute Coronary Syndromes during the COVID-19 pandemic. *Eur Heart J*. 2020;41(19):1839-1851. doi:10.1093/eurheartj/ehaa381 | Donning  1. Remove personal items  2. lead apron  3. First disposable gown  4. Hand wash  5. N95/ FFP2  6. Hair cover  7. Shoe covers  8. Goggles/ Face Shield  9. Hand wash  10. Inner pair of gloves  11. Gown  12. Outer gloves  Doffing  *Inside cath lab*  1. Hand wash  2. Remove gown  3. Remove outer gloves  4. Hand wash  5. Remove face shield/ goggles  6. Hand wash  7. Remove cap/ hair cover  8. Remove shoe covers  9. Hand wash  10. Remove inner gloves  11. Hand wash  *Outside Cath lab*  12. Place additional gloves  13. Remove N95/ FFP2  14. Remove gloves  15. Remove lead apron  16. Hand wash |
| Eid-Lidt | Pubmed accessed on July 15, 2020  Key terms “Cardiac Catheterization PPE” and “COVID and heart catheterization” and “COVID and cardiac catheterization”  37 results  3 with protocols after duplicates were removed | Eid-Lidt G, Farjat Pasos JI. Patient care protocols and personal safety measures for health care professionals in cardiac catheterization rooms during the COVID-19 outbreak in the National Institute of Cardiology [published online ahead of print, 2020 Jun 1]. *Catheter Cardiovasc Interv*. 2020;10.1002/ccd.28979. doi:10.1002/ccd.28979 | Donning  *Outside cath lab*  1. Place disposable uniform  2. Hand wash  3. Lead placement  4. Hand wash  5. Shoe cover/ boots  6. N95 mask  7. Surgical mask  8. Goggles  9. Cap  10. Hand wash  11. Inner gloves  12. Gown  *Inside cath lab*  13. Outer gloves  Doffing  *Inside Cath lab*  1. Remove gown  2. Remove outer glove  3. Hand wash  4. Boot/ shoe removal  5. Hand wash  *Outside Cath lab*  6. Cap removal  7. Wash hands  8. Remove goggles  9. Hand wash  10. Surgical Mask removed  11. Hand wash  12. Remove lead  13. Hand wash  14. Remove other uniform  15. Hand wash  16. Remove inner gloves  17. Hand wash  18. Remove N95 |

**Supplemental Table 2. Number of doffing steps recommended inside versus outside the catheterization laboratory**

| **Institution/ Author/ Society** | **# of Steps Inside Cath Lab** | **# of Steps Outside Cath Lab** |
| --- | --- | --- |
| Sripal Bangalore[8] | 8 | 7 |
| University Health System, San Antonio[9] | 12 | 1 |
| SCAI ELM[10] | 12 | 5 |
| GISE[11] | 12 | 6 |
| Spanish Society of Cardiology[12] | 2 | 6 |
| European Society of Cardiology[13] | Did not designate | Did not designate |
| John, Thadathilankal-Jess et al (Stellenbosch University & Tygerberg Academic Hospital)[14] | Did not designate | Did not designate |
| Indiana American College of Cardiology[15] | 10 | 5 |
| Cardiovascular Innovations Foundation[1] | 11 | 5 |
| Cardiac Society of Australia and New Zealand[16] | 2 | 6 |
| American College of Cardiology: 10 Points to Remember[17] | Did not designate | Did not designate |
| Hammersmith Hospital[18] | 7 | 4 |
| Immad Sadiq[19] | 11 | 15 |
| St. George’s Hospital[20, 21] | 9 | 7 |
| Princess Fatima Academy[22] | Did not designate | Did not designate |
| Giuseppe Tarantini | 11 | 5 |
| CathLab Digest- Morton J. Kern [23] | 0 | 14 |
| International Journal of Cardiovascular Sciences[24] | 8 | 6 |
| SCAI-Jara et. al.[25] | 11 | 6 |
| Bettari et. al.[26] | Did not designate | Did not designate |
| Chieffo et. al.[27] | 11 | 5 |
| Eid Lidt et. al.[28] | 5 | 13 |

**Supplemental Table 3. Patient Mask and Testing Recommendations from the Protocols**

| **Institution/ Author/ Society** | **Recommend Patient Wear a Mask** | **Recommended Testing for COVID** |
| --- | --- | --- |
| NYU/Sripal Bangalore | Did not specify | Did not specify |
| UHS San Antonio | Surgical mask | Test is needed for NSTEMI, individualized plan for STEMI |
| SCAI ELM | Did not specify | Did not specify |
| GISE | Surgical mask | Did not specify |
| Spanish Society of Cardiology | Surgical mask | Do not require testing |
| European Society of Cardiology (Escardio) | Surgical mask | Test for stable NSTEMI, no test needed required for STEMI |
| John, Thadathilankal-Jess et al | Did not specify | Did not specify |
| Indiana American College of Cardiology | Did not specify | Did not specify |
| Cardiovascular Innovations Foundation | Surgical mask | Tested prior to entering cath lab |
| Cardiac Society of Australia and New Zealand | Did not specify | Testing in non-urgent and clinically stable patient |
| American College of Cardiology: 10 Points to Remember | Did not specify | Did not specify |
| Hammersmith Hospital | Did not specify | Did not specify |
| Immad Sadiq | Did not specify | Did not specify |
| St. George’s Hospital | Did not specify | Did not specify |
| Princess Fatima Academy | Surgical mask | Did not specify |
| Giuseppe Tarantini | Surgical mask | Tested prior to enteric cath lab |
| CathLab Digest- Morton J. Kern | Did not specify | Did not specify |
| International Journal of Cardiovascular Sciences | Surgical mask | Did not specify |
| Jara et. al. | Did not specify | Did not specify |
| Bettari et. al. | Surgical mask | Tested positive (case report) |
| Chieffo et. al. | Surgical mask | Test if stable, do not delay if STEMI |
| Eid Lidt et. al. | Did not specify | Did not specify |

**Supplemental Table 4. Number of Hand Washing in Doffing Protocols**

**NOTE: Hand washing includes non-surgical scrub hand wash and surgical scrub hand wash.**

| **Institution/ Author/ Society** | **# of Hand Washing** | **When in the protocol** |
| --- | --- | --- |
| NYU/Sripal Bangalore | 7 | After each removal of PPE |
| Univeristy Health System, San Antonio | 2 | After placement of new gloves |
| SCAI ELM | 5 | Initial, removal of outer gown, removal of inner gown, outside cath lab |
| GISE | 6 | Initial, after removal of outer gloves, removal of shoes, removal of inner gloves, final |
| Spanish Society of Cardiology | 2 | Removal of gown/outer gloves, cap, removal of other PPE |
| European Society of Cardiology | 5 | After each step |
| John, Thadathilankal-Jess et al | 7 | Initial, after each step |
| Indiana American College of Cardiology | 5 | Initial, after removal of outer gown, after removal of gown, outside cath lab, head gear |
| Cardiovascular Innovations Foundation | 6 | Initial, after removal of outer glove, after removal of face shield, after removal of shoe covers, after removal of internal gloves, after removal of lead |
| Cardiac Society of Australia and New Zealand | 2 | Removal of gown/outer gloves, cap, removal of other PPE |
| American College of Cardiology: 10 Points to Remember | 7 | Initial, after removal of shoe covers, after removal of face shield, after removal of outer gown, after removal of outer head cover, after removal of lead, after removal of N95 |
| Hammersmith Hospital | 2 | After removal of lead and other equipment and again after changing |
| Immad Sadiq | 8 | After removal of surgical mask, after removal of outer head cover, after removal of shoe cover, after removal of each pair of gloves |
| St. George’s Hospital | 2 | After removal of both gowns and gloves, final |
| Princess Fatima Academy | 5 | Initial, after removal of outer gown/gloves, after removal of shield, after removal of head cover, after removal of N95 |
| Giuseppe Tarantini | 6 | Initial, after removal of outer glove/gown, after removal of face shield, after removal of shoe cover, after removal of inner gloves, final |
| CathLab Digest- Morton J. Kern | 6 | After removal of shoe covers, after removal of outer gloves, after removal of face shield, after removal of N95, after removal of head cover, after removal of inner glove |
| International Journal of Cardiovascular Sciences | 5 | Initial, after removal of outer glove, after removal of goggles, after removal of N95, final |
| Jara et. al. | 5 | Initial, after removal of outer gloves, after removal of inner gloves, after removal of shoe covers, after removal of inner head cover |
| Bettari et. al. | 0 | Did not designate |
| Chieffo et. al. | 6 | Initial, after removal of outer gloves, after removal of face shield, after removal of shoe covers, after removal of inner gloves, final |
| Eid Lidt et. al. | 8 | After removal of outer glove, after removal of boot/ shoe covers, after every step outside the cath lab |

**Supplemental References**

1. Bangalore S: **Donning and Doffing PPE for the Cath Lab**. In*.*: <https://drive.google.com/file/d/1wPb4eXx1tW5MeuczlZSaxVgWsMBgsqKc/view>.

2. **Cardiology Covid-19 Protocol** [<https://www.universityhealthsystem.com/~/media/files/pdf/covid-19/cardiology-covid-19-protocols-04012020.pdf?la=en>]

3. Szerlip M, Anwaruddin S, Aronow HD, Cohen MG, Daniels MJ, Dehghani P, Drachman DE, Elmariah S, Feldman DN, Garcia S *et al*: **Considerations for cardiac catheterization laboratory procedures during the COVID-19 pandemic perspectives from the Society for Cardiovascular Angiography and Interventions Emerging Leader Mentorship (SCAI ELM) Members and Graduates**. *Catheter Cardiovasc Interv* 2020, **96**(3):586-597.

4. Tarantini G, Fraccaro C, Chieffo A, Marchese A, Tarantino FF, Rigattieri S, Limbruno U, Mauro C, La Manna A, Castiglioni B *et al*: **Italian Society of Interventional Cardiology (GISE) position paper for Cath lab-specific preparedness recommendations for healthcare providers in case of suspected, probable or confirmed cases of COVID-19**. *Catheter Cardiovasc Interv* 2020.

5. Romaguera R, Cruz-González I, Jurado-Román A, Ojeda S, Fernándz-Cisnal A, Jorge-Pérez P, Burgos-Palacios V, Ariza-Solé A, López-de-Sa E, Moreno R: **Considerations on the invasive management of ischemic and structural heart disease during the COVID-19 coronavirus outbreak**. *REC Interv Cardiol* 2020.

6. **European Society of Cardiology Guidance for the Diagnosis and Management of CV Disease during the COVID-19 Pandemic**. 2020.

7. John TJ, Hassan K, Weich H: **Donning and doffing of personal protective equipment (PPE) for angiography during the COVID-19 crisis**. *Eur Heart J* 2020, **41**(19):1786-1787.

8. **Cath Lab Protocols for COVID-19**.

9. Lo STH, Yong AS, Sinhal A, Shetty S, McCann A, Clark D, Galligan L, El-Jack S, Sader M, Tan R *et al*: **Consensus guidelines for interventional cardiology services delivery during covid-19 pandemic in Australia and new Zealand**. *Heart Lung Circ* 2020, **29**(6):e69-e77.

10. Mukherjee D: **American College of Cardiology: Ten Points to Remember for Donning and Doffing of Personal Protective Equipment for Angiography**.

11. **Protect the Professional: Personal Protection Equipment (PPE) in the Cath lab during COVID-19-Hammersmith Hospital**.

12. Sadiq I: **COVID-19 CathLab Donning and Doffing Protocol video**.

13. **ST George's Hospital Cath Lab Pathway for COVID-19 Prepared Labs PART-1**.

14. **St Georges Cath Lab Pathway for COVID-19 Prepared Labs PART-2**.

15. Ahmed A: **Lecture #16: How to Prepare your Cathlab Structure for COVID - 19 Patients_Part II**.

16. Kern MJ: **“The D’Officer” – Tips on Donning and Doffing PPE**.

17. Mariano GZ, Lemke VG, Paiva MSM, Oliveira GMMd: **Covid-19 and Safety in the Cath Lab: Where We Are and Where We Are Headed**. *International Journal of Cardiovascular Sciences* 2020, **33**:288-294.

18. Cesar Jara BS, Rajesh Swaminathan, Jayant Bagai, Faial Latif, Kirk Garratt, Ehtisham Mahmud: **PPE Donning and Doffing in Cardiac Catheterization Labs During the COVID-19 Pandemic**. 2020.

19. Bettari L, Pero G, Maiandi C, Messina A, Saccocci M, Cirillo M, Troise G, Conti E, Cuccia C, Maffeo D: **Exploring Personal Protection During High-Risk PCI in a COVID-19 Patient: Impella CP Mechanical Support During ULMCA Bifurcation Stenting**. *JACC: Case Reports* 2020, **2**(9):1279-1283.

20. Chieffo A, Stefanini GG, Price S, Barbato E, Tarantini G, Karam N, Moreno R, Buchanan GL, Gilard M, Halvorsen S *et al*: **EAPCI Position Statement on Invasive Management of Acute Coronary Syndromes during the COVID-19 pandemic**. *European Heart Journal* 2020, **41**(19):1839-1851.

21. Eid-Lidt G, Farjat Pasos JI: **Patient care protocols and personal safety measures for health care professionals in cardiac catheterization rooms during the COVID-19 outbreak in the National Institute of Cardiology**. *Catheterization and Cardiovascular Interventions*, **n/a**(n/a).
